# Vessel and balloon sizing in the IN.PACT AV access trial: post-hoc analysis of procedural characteristics and outcomes

**DOI:** 10.1186/s42155-026-00650-6

**Published:** 2026-02-14

**Authors:** Andrew Holden, Hiroaki Haruguchi, Kotaro Suemitsu, Naoko Isogai, Jeffrey Hull, Bret N. Wiechmann, Hong Wang, Bridget Wall, Robert Lookstein, IN.PACT AV Access Investigators, Andrew Holden, Kotaro Suemitsu, Naoko Isogai, Jeffrey Hull, Bret N. Wiechmann, Levester Kirksey, Federico Parodi, David Hardy, Sean Lyden, Sanjay Misra, Haraldur Bjarnason, Andrew Stockland, Newton Neidert, Emily Bendel, Christopher Reisenauer, Melissa Neisen, Erica Knavel, Angelo Santos, Chad Laurich, Patrick Kelly, Omran Abul-Khoudoud, Alexander Hou, Paul Lewis, Adie Friedman, Michael Dudkiewicz, Ronald Dreifuss, John Rundback, Kevin Herman, Husameddin El Khudari, Ahmed Kamel Abdel Aal, Nathan Ertel, Rachel Oser, Andrew Gunn, Mel Sharafuddin, Sandeep Laroia, Shiliang Sun, Brendan O’Shea, Brian Miller, Timothy Kresowik, William Sharp, Shengfu Wang, Sreekumar Madassery, David Dexter, Samuel Steerman, Animesh Rathore, Richard DeMasi, Gordon Stokes, Scott McEnroe, Charles Joels, Mark Fugate, Michael Greer, Larry Richard Sprouse, Jeff Horn, Syed Hussain, Nikolaos Karagiorgos, Jennifer Ash, Sandeep Bagla, Rachel Piechowiak, Mark London, John Ross, Jackson Ewart, Jalal Hakmei, Mohamed Sheta, Jeffrey Hoggard, Karn Gupta, Sejan Patel, Wesley Mann, Naveen Atray, Rohit Kashyap, Karthik Ramani, Randy Cooper, Aslam Pervez, Umar Waheed, Neghae Mawla, Steven Beathard, Fernando Kafie, Huey McDaniel, Yuki Matsuoka, Naomi Ota, Kanako Oka, Saho Kawanishi, Hidemitsu Ogino, Katsunori Miyake, Jun Kawachi, Takaaki Murata, Nao Kume, Yuto Igarashi, Yuma Sunou, Sumi Hidaka, Kunihiro Ishioka, Masahiko Fujihara, Yoshiaki Yokoi, Akihiro Higashimori, Nobuyuki Morioka, Shinji Shiotani, Keisuke Fukuda, Tomofumi Tsukizawa, Kensuke Kuwabara, Yoshiki Matsuo, Yuma Tanabe, Masaaki Murakami, Noriko Mori, Kiyoshi Mori, Satoshi Tanaka, Ken Matsuo, Takao Okawa, Shunsuke Okamura, Yu Soma, Yoshihiro Yamamoto, Shota Kimura, Yuki Ito, Akira Sugawara, Kenta Ito, Ryo Yamada, Yoko Matsuo, Kakuya Hagiwara, Kazuhiro Iwadoh, Ichiro Nakajima, Shohei Fuchinoue, Sachiko Hirotani, Kotaro Kai, Yuichi Ogawa, Katsuyuki Miki, Takeshi Hachisuka, Sayaka Morita, Akira Kondo, Kazuhiro Iwadoh, Tomonari Ogawa, Hajime Hasegawa, Toru Hida, Taisuke Shimizu, Nozomi Abe, Tatsuro Sano, Kunihiko Yasuda, Tota Kiba, Yoshimi Okada, Koki Ogawa, Hiroaki Hara, Kento Hirose, Yuichiro Kawai, Brendan Buckley, Stephen Merrilees, David Semple, Andrew Hill, Janaka Kesara Wickremesekera, Richard Evans, Lupe Taumoepeau, Anantha Narayanan, Irina Baimatova

**Affiliations:** 1 Department of Interventional Radiology, Auckland Hospital, Auckland, New Zealand; 2 Haruguchi Vascular Access Clinic, Tokyo, Japan; 3 Osaka Keisatsu Hospital, Osaka, Japan; 4 Shonan Kamakura General Hospital, Kanagawa, Japan; 5 Richmond Vascular Center, Richmond, VA USA; 6 Vascular and Interventional Physicians, Gainesville, FL USA; 7 Medtronic, Plymouth, MN USA; 8 Department of Diagnostic, Molecular, and Interventional Radiology at the Icahn School of Medicine at Mount Sinai, New York City, NY USA

**Keywords:** End stage renal disease, Peripheral vascular, Angioplasty, Angiogram, Dialysis access, Dialysis, Hemodialysis, Revascularization, Revascularisation

## Abstract

**Background:**

Drug-coated balloons (DCBs) have demonstrated effectiveness and safety in the treatment of dysfunctional arteriovenous fistulas used for hemodialysis in larger randomized studies; however, the patient and lesion profiles that have the best DCB outcomes remain undefined. Pivotal trials with core lab adjudication are ideal to generate hypotheses for future studies of intra-procedural characteristics given they include both site-reported and independently assessed data.

**Methods:**

The IN.PACT AV Access Trial randomized 330 patients 1:1 to treatment with a DCB (n = 170) or uncoated percutaneous transluminal angioplasty (PTA; n = 160). This exploratory post-hoc analysis investigated core lab adjudicated vessel sizing in the context of target lesion primary patency (TLPP) outcomes through 36 months. Participants were split into groups by median reference vessel diameter (RVD).

**Results:**

Site-reported and core lab adjudicated RVDs were different (7.24 ± 2.00 vs 7.62 ± 2.39 mm). Approximately two-thirds of balloons were over- or under-sized (*n*=219). DCB demonstrated significantly improved TLPP through 36 months in both vessel size subgroups compared to PTA (<7.17 mm 42.2% DCB (*n*=84) vs 28.6% PTA (*n*=81), log-rank *p*=0.024; ≥7.17 mm 43.9% DCB (*n*=86) vs 28.7% PTA (*n*=79), log-rank *p*=0.011).

**Conclusions:**

This post hoc analysis identified that intra-procedural sizing decisions have the potential to positively impact outcomes in patients with dysfunctional fistula lesions. These findings warrant further prospective evaluation to define optimal DCB sizing strategies.

**Trial registration:**

NCT03041467. Registered 25 April 2017.

**Level of evidence:**

3

**Graphical Abstract:**

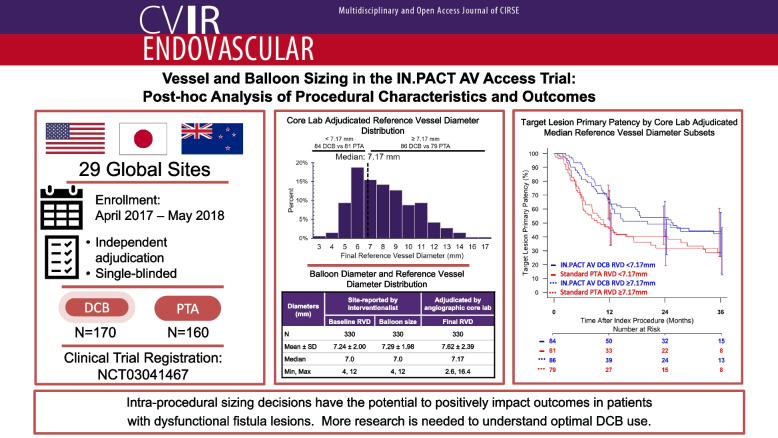

**Supplementary Information:**

The online version contains supplementary material available at 10.1186/s42155-026-00650-6.

## Background

Treatment with drug-coated balloon (DCB) angioplasty is associated with improved short and mid-term patency compared to uncoated percutaneous transluminal angioplasty (PTA) when used to treat dysfunctional arteriovenous fistulas (AVF) [1, 2]. As use of DCBs has increased and data has continued to be reported in the peer-reviewed literature, a common question is “when, where, and with which patient is a DCB most effective?” Sub-analyses from larger multicenter trials have begun to define these optimal situations [3]; however, an important gap remains: even if clinical trial data defines the right time and place to use DCB, if standard practice does not follow the same intraprocedural steps, outcomes in a real-world population may not be as positive.

This post-hoc analysis of a study that included both site-reported and core lab adjudicated data seeks to generate hypotheses for future studies regarding vessel sizing and subsequent balloon sizing choices to optimize clinical outcomes in this difficult-to-treat patient population and help define where DCB is most effective.

## Methods

The IN.PACT AV Access trial enrolled participants at 29 sites in 3 countries (USA, Japan, and New Zealand; Supplementary Table S1). Participants eligible to join the trial based on inclusion and exclusion criteria were randomized 1:1 to treatment with an IN.PACT AV DCB (Medtronic; Plymouth, MN; *n* = 170) or standard uncoated PTA (*n* = 160). The full protocol and methods have been previously reported in full.

Briefly, this trial was registered as NCT03041467 with ClinicalTrials.gov and was conducted in accordance with the Declaration of Helsinki. Ethics committees or institutional review boards reviewed and approved trial protocols and amendments. Data were analyzed by Medtronic and the Baim Institute for Clinical Research (Boston, Massachusetts). This trial was originally designed to go through 24 months of follow-up; however, the trial was extended out to 60 months after concerns about the safety of the paclitaxel coating were raised in late 2018. There is a subset of patients that did not reconsent, as shown in the consort diagram published through 36 months [4]. The data that support the findings of this study are available from the corresponding author upon reasonable request. The study sponsor (Medtronic) will evaluate on a study-by-study basis whether there is an opportunity to share clinical trial data with qualified scientific or medical researchers, consistent with the associated informed consent and applicable privacy laws and regulations.

Participants were enrolled and randomized following successful predilation with a high-pressure balloon, which rested on an interventionalist’s clinical judgement that the lesion reached a residual stenosis ≤ 30%. This residual stenosis and all other vessel sizing measurements were also adjudicated by the angiographic core laboratory (Synactx, New York, NY, USA) after the index procedure had been completed.

Target lesion primary patency (TLPP), the primary outcome of which occurred at 6 months, was evaluated through the 36-month timepoint. Loss of TLPP occurred if a participant had an access circuit thrombosis or required a target lesion revascularization (TLR).

### Statistical analysis

**Fig. 1:**
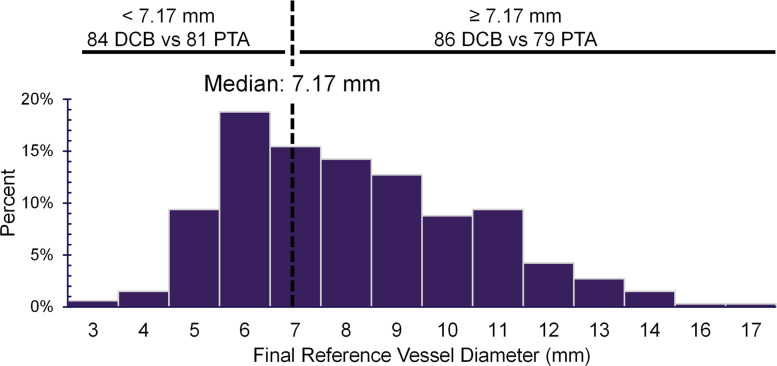
Distribution of core lab adjudicated final reference vessel diameter, DCB, drug-coated balloon; PTA, percutaneous transluminal angioplasty

In this post-hoc analysis, TLPP was reported by two sets of procedural characteristics: vessel size and balloon size. For the vessel size analysis, the median final reference vessel diameter (RVD) evaluated by the angiographic core lab at the index procedure was used to split participants into groups of RVD < 7.17 mm vs ≥ 7.17 mm; Fig. 1. The distribution is right-skewed. In the group with RVDs less than 7.17 mm (*n* = 84 DCB, *n* = 81 PTA), most participants typically fell within the 6 to 7.17 mm range. Conversely, participants in the group with RVDs of 7.17 mm or greater (*n* = 86 DCB, *n* = 79 PTA) were primarily distributed between 7.17 mm and 10 mm, with the remainder spread across higher RVDs.

A complementary balloon size analysis was included in the supplement; the site-reported median of the maximum treatment balloon diameter for each participant at the index procedure was used to split participants into groups of balloon diameters < 7 mm (*n* = 78 DCB, *n* = 70 PTA) and ≥ 7 mm (*n* = 92 DCB, *n* = 90 PTA; Supplementary Fig. S1). The distribution was right skewed.

**Fig. 2:**
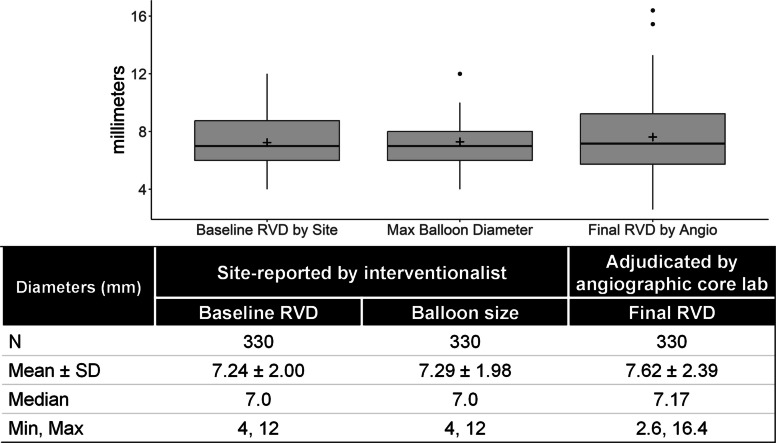
Balloon diameters and reference vessel diameter distribution. DCB, drug-coated balloon; PTA, percutaneous transluminal angioplasty; RVD, reference vessel diameter

Balloon diameters and the site-reported versus core lab adjudicated RVDs were summarized: the mean baseline RVD by site was 7.24 ± 2.00 mm, the mean balloon size by site was 7.29 ± 1.98 mm, and the mean final RVD adjudicated by the core laboratory was 7.62 ± 2.39 mm (Fig. 2). This mismatch between measurements led to the clinical question about over- and under-sizing of balloons compared to the adjudicated size of the vessel. Balloon sizes were compared to the adjudicated final RVD; approximately two thirds of balloons were over-sized or under-sized in this study (Supplementary Fig. S2).

Analyses were based on randomization assignment with evaluable data (intent-to-treat) within each stratified treatment balloon size and vessel subgroups respectively. Descriptive statistics were provided for baseline demographics, disease, lesion, and procedural characteristics. Continuous variables are summarized as mean ± SD, and dichotomous or categorical variables as percentages and counts. Continuous variables were compared with the independent *t* test, and dichotomous and categorical variables using Fisher exact test or Cochran-Mantel–Haenszel modified ridit scores, respectively. Time to event outcomes were analyzed using the Kaplan–Meier method; confidence intervals (95% CI) were computed for time-to-event outcomes using the log–log transformation; differences between survival curves were assessed using log-rank tests. Participants were censored at the time of AVF abandonment, death, loss-to-follow-up, or withdrawal from the study. Cox proportional hazards models were used to estimate hazard ratios (HR) and 95% confidence intervals comparing treatment groups for each endpoint within each subgroup, respectively. Annual cutoffs for the statistical analysis used 360 days per year (e.g., 1080 days for the 3-year cut-off). No adjustment was made for multiple comparisons, and the level of statistical significance was set at 0.05. Given the exploratory, post-hoc nature of the analyses, all reported p-values should be interpreted as descriptive rather than inferential. Statistical analyses were performed using SAS version 9.4 (SAS Institute, Cary, NC, USA).

## Results

Baseline and procedural characteristics of the full cohort have been previously reported [4–6]; baseline and procedural characteristics of the groups stratified by RVD are reported in Table 1.

**Table 1:** Baseline demographics and procedural characteristics by core lab adjudicated RVD

Baseline participant and lesion characteristics^a^	Reference vessel diameter < 7.17 mm				Reference vessel diameter ≥ 7.17 mm			
	**IN.PACT AV DCB** *N*** = 84**	**Standard PTA** *N*** = 81**	**Total** *N*** = 165**	*p***-value**^**b**^	**IN.PACT AV Access DCB** *N*** = 86**	**Standard PTA** *N*** = 79**	**Total** *N*** = 165**	*p***-value**^**c**^
Age (years)	68.5 ± 12.1 (84)	67.2 ± 12.9 (81)	67.8 ± 12.5 (165)	0.504	63.2 ± 13.6 (86)	63.7 ± 13.9 (79)	63.4 ± 13.7 (165)	0.781
Male	66.7% (56/84)	64.2% (52/81)	65.5% (108/165)	0.746	65.1% (56/86)	62.0% (49/79)	63.6% (105/165)	0.747
Hypertension	89.3% (75/84)	93.8% (76/81)	91.5% (151/165)	0.404	93.0% (80/86)	94.9% (75/79)	93.9% (155/165)	0.749
Hyperlipidemia	59.5% (50/84)	48.1% (39/81)	53.9% (89/165)	0.161	48.8% (42/86)	57.0% (45/79)	52.7% (87/165)	0.350
Diabetes Mellitus	63.1% (53/84)	69.1% (56/81)	66.1% (109/165)	0.511	62.8% (54/86)	68.4% (54/79)	65.5% (108/165)	0.513
Renal Insufficiency	100.0% (84/84)	100.0% (81/81)	100.0% (165/165)	> 0.999	100.0% (86/86)	100.0% (79/79)	100.0% (165/165)	> 0.999
Carotid Artery Disease	3.6% (3/84)	4.9% (4/81)	4.2% (7/165)	0.717	4.7% (4/86)	12.7% (10/79)	8.5% (14/165)	0.093
Congestive Heart Failure	22.6% (19/84)	21.0% (17/81)	21.8% (36/165)	0.852	23.3% (20/86)	27.8% (22/79)	25.5% (42/165)	0.592
Coronary Heart Disease	41.7% (35/84)	42.0% (34/81)	41.8% (69/165)	1.000	30.2% (26/86)	35.4% (28/79)	32.7% (54/165)	0.510
Peripheral Artery Disease	22.6% (19/84)	16.0% (13/81)	19.4% (32/165)	0.328	16.3% (14/86)	14.1% (11/78)	15.2% (25/164)	0.828
Current Smoker	15.5% (13/84)	14.8% (12/81)	15.2% (25/165)	1.000	7.0% (6/86)	17.7% (14/79)	12.1% (20/165)	0.054
Former Smoker	39.3% (33/84)	27.2% (22/81)	33.3% (55/165)	0.137	34.9% (30/86)	29.1% (23/79)	32.1% (53/165)	0.505
Lesion Type				0.863				1.000
De Novo	27.4% (23/84)	29.6% (24/81)	28.5% (47/165)		32.6% (28/86)	31.6% (25/79)	32.1% (53/165)	
Restenotic	72.6% (61/84)	70.4% (57/81)	71.5% (118/165)		67.4% (58/86)	68.4% (54/79)	67.9% (112/165)	
Lesion classification				0.159				0.438
Single	77.4% (65/84)	86.4% (70/81)	81.8% (135/165)		88.4% (76/86)	92.4% (73/79)	90.3% (149/165)	
Tandem	22.6% (19/84)	13.6% (11/81)	18.2% (30/165)		11.6% (10/86)	7.6% (6/79)	9.7% (16/165)	
Target Lesion Location				0.547				0.694
Anastomosis	42.9% (36/84)	43.2% (35/81)	43.0% (71/165)		9.3% (8/86)	6.3% (5/79)	7.9% (13/165)	
Arterial Inflow	2.4% (2/84)	6.2% (5/81)	4.2% (7/165)		2.3% (2/86)	2.5% (2/79)	2.4% (4/165)	
Cephalic Arch	4.8% (4/84)	6.2% (5/81)	5.5% (9/165)		30.2% (26/86)	39.2% (31/79)	34.5% (57/165)	
In Cannulation Zone	11.9% (10/84)	4.9% (4/81)	8.5% (14/165)		17.4% (15/86)	10.1% (8/79)	13.9% (23/165)	
Swing Point	7.1% (6/84)	6.2% (5/81)	6.7% (11/165)		9.3% (8/86)	8.9% (7/79)	9.1% (15/165)	
Venous Outflow	31.0% (26/84)	33.3% (27/81)	32.1% (53/165)		31.4% (27/86)	32.9% (26/79)	32.1% (53/165)	
AVF Type				0.914				0.915
Radiocephalic	78.6% (66/84)	77.8% (63/81)	78.2% (129/165)		23.3% (20/86)	21.5% (17/79)	22.4% (37/165)	
Brachiocephalic	15.5% (13/84)	13.6% (11/81)	14.5% (24/165)		57.0% (49/86)	59.5% (47/79)	58.2% (96/165)	
Brachiobasilic	2.4% (2/84)	3.7% (3/81)	3.0% (5/165)		17.4% (15/86)	15.2% (12/79)	16.4% (27/165)	
Other	3.6% (3/84)	4.9% (4/81)	4.2% (7/165)		2.3% (2/86)	3.8% (3/79)	3.0% (5/165)	
Previous peripheral revascularization	77.4% (65/84)	76.5% (62/81)	77.0% (127/165)	1.000	70.9% (61/86)	73.4% (58/79)	72.1% (119/165)	0.732
Time since AVF creation (years)^d^	3.1 ± 3.0 (84)	3.7 ± 4.5 (81)	3.4 ± 3.8 (165)	0.373	3.3 ± 3.0 (86)	3.3 ± 3.0 (79)	3.3 ± 3.0 (165)	0.920
Time on hemodialysis (years) ^e^	4.7 ± 5.8 (84)	4.6 ± 6.4 (81)	4.6 ± 6.1 (165)	0.910	4.0 ± 4.3 (86)	3.8 ± 3.5 (78)	3.9 ± 3.9 (164)	0.663
Total lesion length (mm)	57.7 ± 28.4 (84)	51.6 ± 25.5 (81)	54.7 ± 27.1 (165)	0.149	36.3 ± 23.4 (86)	28.0 ± 20.0 (79)	32.3 ± 22.2 (165)	0.016
Pre-procedure stenosis (percent)	83.1 ± 12.6 (84)	81.5 ± 13.5 (81)	82.3 ± 13.1 (165)	0.429	77.5 ± 11.1 (86)	76.8 ± 11.4 (79)	77.2 ± 11.2 (165)	0.689

^a^ % (counts/sample size) or mean ± standard deviation (sample size)

^b^ *P* values in this column are comparing DCB to PTA outcomes in those participants with reference vessel diameters < 7.17 mm

^c^ *P* values in this column are comparing DCB to PTA outcomes in those participants with reference vessel diameters ≥ 7.17 mm

^d^24 participants had partial dates and their years since AVF creation were calculated based on the imputed dates using the middle of the month or middle of the year

^e^29 participants had partial dates and their years of hemodialysis history were calculated based on the imputed dates using the middle of the month or middle of the year

**Fig. 3:**
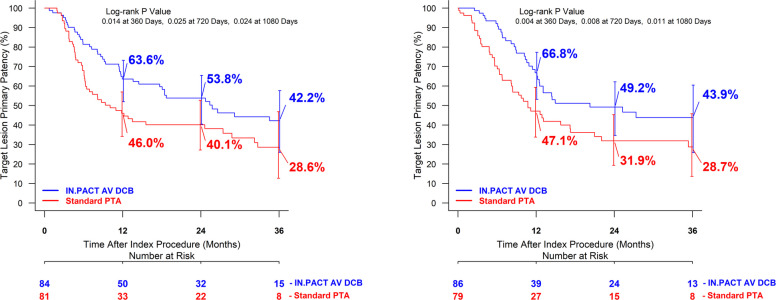
**a**, **b** Target lesion primary patency through 36 months by core lab adjudicated final median reference vessel diameter (A, < 7.17 mm; B, ≥ 7.17 mm). DCB, drug-coated balloon; PTA, percutaneous transluminal angioplasty. All events were adjudicated by the independent and blinded Clinical Events Committee. Target lesion primary patency is defined as freedom from clinically-driven target lesion revascularization or access circuit thrombosis. An event was adjudicated as a clinically-driven target lesion revascularization if the target lesion had a ≥50% diameter stenosis (per angiographic core lab assessment) in the presence of clinical or physiologic abnormalities that indicate dialysis access dysfunction or a ≥70% stenosis without the presence of clinical or physiologic abnormalities indicating dialysis access dysfunction

TLPP using Kaplan–Meier analysis demonstrated a significant difference between DCB and PTA within each vessel size subgroup (RVD < 7.17 mm and RVD ≥ 7.17 mm) at 36 months (Fig. 3a, b; Supplementary Figs. S3 and S4; Supplementary Table S2). Complementary analyses investigating balloon size are reported in Supplementary Table S3 and Supplementary Figs. S5 and S6.

**Fig. 4:**
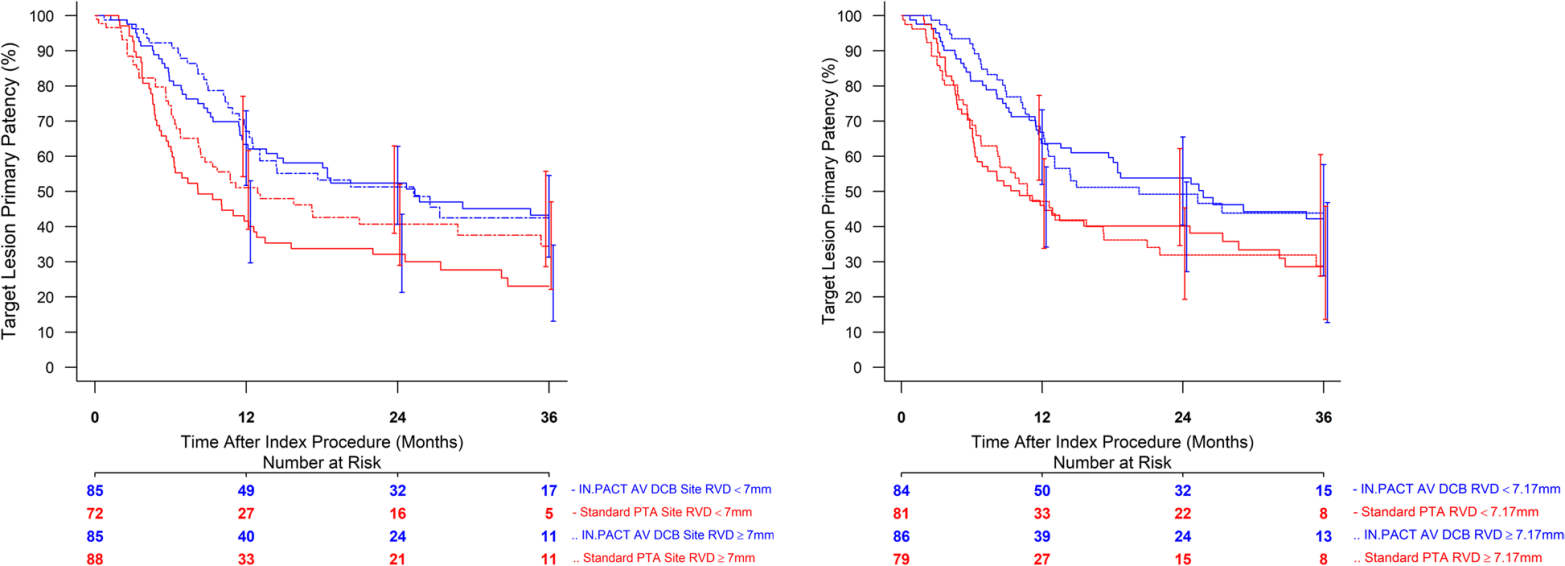
**a**, **b** Target lesion primary patency through 36 months comparing site-reported RVD and core lab adjudicated RVD outcomes stratified by RVD median (A, site-reported; B, core lab adjudicated). DCB, drug-coated balloon; PTA, percutaneous transluminal angioplasty. All events were adjudicated by the independent and blinded Clinical Events Committee. Target lesion primary patency is defined as freedom from clinically-driven target lesion revascularization or access circuit thrombosis. An event was adjudicated as a clinically-driven target lesion revascularization if the target lesion had a ≥50% diameter stenosis (per angiographic core lab assessment) in the presence of clinical or physiologic abnormalities that indicate dialysis access dysfunction or a ≥70% stenosis without the presence of clinical or physiologic abnormalities indicating dialysis access dysfunction

When outcomes are compared by site-reported RVD and core lab adjudicated RVD, DCB performance was similar in all groups (Fig. 4a, b). However, in the site-reported analysis, PTA outcomes were improved in large vessels.

**Fig. 5:**
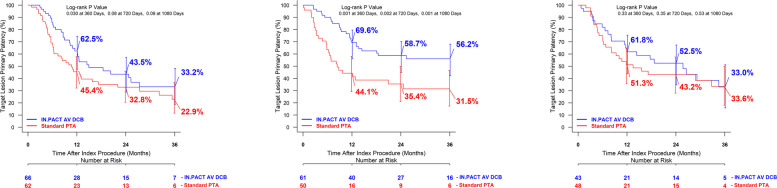
**A**, **B**, and **C** Target lesion primary patency through 36 months comparing site-reported balloon sizes and core lab adjudicated RVD (**A**, undersized by at least 0.5 mm; **B**, insize within 0.5 mm; **C** oversized by at least 0.5 mm). DCB, drug-coated balloon; PTA, percutaneous transluminal angioplasty. All events were adjudicated by the independent and blinded Clinical Events Committee. Target lesion primary patency is defined as freedom from clinically driven target lesion revascularization or access circuit thrombosis. An event was adjudicated as a clinically driven target lesion revascularization if the target lesion had a ≥ 50% diameter stenosis (per angiographic core lab assessment) in the presence of clinical or physiologic abnormalities that indicate dialysis access dysfunction or a ≥ 70% stenosis without the presence of clinical or physiologic abnormalities indicating dialysis access dysfunction

A further hypothesis-generating Kaplan–Meier analysis demonstrated that DCB sized within 0.5 mm of the core lab adjudicated RVD had the best outcomes throughout the trial (Fig. 5a–c).

## Discussion

Treatment of dysfunctional arteriovenous fistulas used for hemodialysis continues to be challenging: levels of restenosis are so high in real-world populations that the current goal of the KDOQI guidelines released in 2019 states that “each fistula should have fewer than 3 interventions per year” [7]. The rates of patency with treatment using PTA are reported in meta-analyses to be 50–60% at 6 months and 10–25% at 24 months [8, 9]. DCBs have been associated with improved short and mid-term patency in early clinical use and subsequently in larger randomized controlled trials [1, 2]. The current open question is optimal DCB use, [2] especially given the additional cost of these devices [10].

As intraprocedural choices are under the control of the interventionalist, they are an important consideration as optimal use of DCBs is defined. There has been limited research conducted on intraprocedural characteristics and downstream outcomes in this patient population; they have mainly focused on balloon inflation time and pressure, and were conducted on hemodialysis access grafts or mixed populations of grafts and fistulas [11–13]. Guidelines for clinical practice or clinical trial endpoints therefore have little to offer in terms of balloon or vessel sizing recommendations for dysfunctional fistula treatment or sizing techniques (Supplementary Table S4) [7, 14–23].

Device instructions for use can provide some information: for the two DCBs approved for use in dysfunctional fistulas in the USA at the time of this publication, Lutonix recommends 1:1 balloon sizing compared to the predilation balloon, and IN.PACT AV DCB recommends 1:1 vessel:balloon sizing [24, 25]. Specific instructions were also included within their investigational device exemption (IDE) studies. In the IN.PACT AV Access study discussed in this manuscript, the protocol specified that “balloon size must match the RVD distal to the target lesion” [5]. In the Lutonix AV study, the protocol specified that “study and control balloon diameters were identical to that of the high pressure predilation balloon, the diameter of which was selected at the investigator’s discretion” [26].

Outcomes by intraprocedural characteristics were not reported in the Lutonix AV IDE [3] or the PAVE study, [27] but they were reported in the real-world Lutonix Global AV Registry. This multicenter prospective study of 320 participants treated 392 lesions with the Lutonix DCB [28]. Both predilation and longer inflation time increased patency outcomes through 6 months [28]. Both predilation and a 3-min inflation time were required in the IN.PACT AV Access trial and likely contributed to the improved outcomes in the DCB group at all timepoints.

In the IN.PACT AV Access study reported here, RVD was noted by the interventionalist during the procedure, and then images were adjudicated by a core laboratory. These RVD measurements were different when measured by interventionalists compared to the core laboratory. This discrepancy could potentially originate from interventionalist bias, technical bias, or both. Interventionalist biases include training background, the enrolling hospital’s standard of care, potential decisions to ‘round up’ or ‘round down’ sizing for ease of balloon sizing estimations, amount of contrast usage, and previous knowledge of the patient’s imaging. Technical biases include the type of software, calibration mode and frequency, vessel size impacting ability to appropriately measure (e.g., a larger vessel is easier to measure more accurately), and the level of image magnification. This led to a balloon-to-vessel mismatch in approximately two thirds of the balloons, with appropriately sized balloons having the largest treatment effect difference between DCB and PTA in this post-hoc analysis. Especially as outcomes appear worse with small balloons but similar with small vessels—this leads to many questions about what is influencing outcomes during a procedure. Open questions of interest include determining whether it is worse to over- or undersize a balloon, determining whether sizing choices of balloons in fistula should be different than in other arterial and venous vascular beds, and which balloon characteristic impacts outcomes the most.

Based on these hypothesis-generating outcomes, we therefore recommend the following when designing future prospective or retrospective studies focused on intraprocedural characteristics affecting outcomes with DCB. First, they could begin with a review of any previous imaging to check vessel and balloon sizing, and how they compare to the current target lesion. During the intervention, strategies to measure RVD include angiographic quantitative vessel analysis software, transcutaneous ultrasound, and image overlay with a semi-compliant pre-dilatation angioplasty balloon. Intra-vascular imaging technologies such as intravascular ultrasound (IVUS) and optical coherence tomography (OCT) are not routinely used due to cost and complexity, but it is possible these could be associated with improved outcomes. There are added complexities in accurate RVD estimation when there is a significant change in diameter between the vessel proximal to the stenosis and the vessel distal to the stenosis (e.g., the arterio-venous anastomosis, the cephalo-subclavian junction, or post-stenotic dilatation).

Once a balloon type is chosen, deployment characteristics could be captured: time of inflation, specific type and brand of modality, device diameter and length, and pressure. If a stenosis is resistant and an ultra high-pressure balloon is needed, those details could be noted as well. After treatment, additional images and use of quantitative measurement software could compare pre- and post-procedure imaging, and note the change in percent residual stenosis. If possible, submission of all images to an independent core laboratory could provide guidance on the differences between a specific site’s judgement of sizing and how those sizes are quantified by an independent observer.

While the particularly rigorous information listed above and independent core lab adjudication are not reasonable to gather within the majority of interventionalist’s daily practice, we note these measurement techniques are device agnostic and may provide patient benefit. Even though the outcomes with DCB in this particular study were statistically significantly better than PTA, outcomes with uncoated balloons were better than typical PTA outcomes, likely due to the careful lesion preparation.

There are two other post-approval trials ongoing that will also ideally expand the understanding of optimal balloon and DCB use in particular: the Lutonix post-approval study (NCT03506308) and the IN.PACT AV Access post-approval study (NCT04543539). Another large study, PAVE-2 (ISRCTN40182296), will compare the IN.PACT AV DCB (paclitaxel) and the MagicTouch DCB (sirolimus) to a plain uncoated balloon control. In addition, we call on individual interventionalists or teams to publish their own longitudinal studies of specific fistula outcomes tied to the myriad procedural characteristics available to add valuable clinical data defining optimal DCB use. 

This was a post-hoc analysis of solely 330 patients studied in a pivotal trial; data is not real-world. There was no way to use core lab adjudicated findings to decide device size choice in this trial. Given the variability inherent to fistula and lesion type, measurement of RVD distal to the target lesion may not be equally meaningful in comparing procedural outcomes for all fistula and lesion types. Additionally, multiple comparisons were performed without adjustment for multiplicity; as such, all reported p-values should be interpreted as descriptive. Future studies may benefit from external third-party statistical review to further enhance objectivity.

## Conclusions

This post-hoc and hypothesis-generating analysis of the IN.PACT AV Access trial demonstrated the potential for intra-procedural sizing decisions to positively impact outcomes in patients with dysfunctional fistula lesions. These findings warrant further prospective evaluation to define optimal DCB sizing strategies.

## Supplementary Information

Supplementary Material 1: Table S1. Investigators

Supplementary Material 2: Table S2. 36-month target lesion primary patency Kaplan-Meier analyses and 95% confidence intervals by balloon size

Supplementary Material 3: Table S3. Baseline demographics + procedural characteristics by balloon size

Supplementary Material 4: Table S4. Summary of guideline recommendations about vessel sizing in the AV space

Supplementary Material 5 Figure S1. Distribution of site-reported balloon diameter

Supplementary Material 6: Figure S2. Site-reported balloon size compared to core lab adjudicated reference vessel diameter

Supplementary Material 7 Figure S3. Forest plot of target lesion primary patency through 36 months by core lab adjudicated median final reference vessel diameter

Supplementary Material 8: Figure S4. Kaplan-Meier analysis of target lesion primary patency through 36 months by balloon diameter (a, <7 mm; b ≥7 mm)

Supplementary Material 9: Figure S5. Kaplan-Meier analysis of target lesion primary patency through 36 months by balloon diameter

Supplementary Material 10: Figure S6. Forest plot of target lesion primary patency through 36 months by balloon diameter

## Data Availability

The data that support the findings of this study are available from the corresponding author upon reasonable request. The study sponsor (Medtronic) will evaluate on a study-by-study basis whether there is an opportunity to share clinical trial data with qualified scientific or medical researchers, consistent with the associated informed consent and applicable privacy laws and regulations.
