# Supplementary material for: Vessel and balloon sizing in the IN.PACT AV access trial: post-hoc analysis of procedural characteristics and outcomes
Source: CVIR Endovasc. 2026 Feb 14;9:17. doi: 10.1186/s42155-026-00650-6 (PMC12906498; doi:10.1186/s42155-026-00650-6)
Supplement: Supplementary file 1 — Supplementary Material 1: Table S1. Investigators [file 42155_2026_650_MOESM1_ESM.pdf]

Supplemental Table 1 – Investigators

Clinical Site – United States	Location	Principal Investigator	Sub-Investigator
Cleveland Clinic	Cleveland, OH	Levester Kirksey, MD	Federico Parodi, MD<br>David Hardy, MD<br>Sean Lyden, MD
Mayo Clinic	Rochester, MN	Sanjay Misra, MD	Haraldur Bjarnason, MD<br>Andrew Stockland, MD<br>Newton Neidert, MD<br>Emily Bendel, MD<br>Christopher Reisenauer, MD<br>Melissa Neisen, MD<br>Erica Knavel, MD
Sanford University of South Dakota (USD) Medical Center	Sioux Falls, SD	Angelo Santos, MD<br>Chad Laurich, MD	Patrick Kelly, MD
King's Daughters Medical Center	Ashland, KY	Omran Abul-Khoudoud, MD	Alexander Hou, MD<br>Paul Lewis, MD
The Mount Sinai Hospital	New York, NY	Adie Friedman, MD	Michael Dudkiewicz, MD<br>Ronald Dreifuss, MD

Holy Name Medical Center	Teaneck, NJ	John Rundback, MD	Kevin Herman, MD
University of Alabama at Birmingham (UAB) Hospital	Birmingham, AL	Husameddin El Khudari, MD<br>Ahmed Kamel Abdel Aal, MD	Nathan Ertel, MD<br>Rachel Oser, MD<br>Andrew Gunn, MD
University of Iowa Hospitals and Clinics	Iowa City, IA	Mel Sharafuddin, MD	Sandeep Laroia, MD<br>Shiliang Sun, MD<br>Brendan O'Shea, MD<br>Brian Miller, MD<br>Timothy Kresowik, MD<br>William Sharp, MD<br>Shengfu Wang, MD
Rush University Medical Center	Chicago, IL	Sreekumar Madassery, MD	
Sentara Vascular Specialists	Norfolk, VA	David Dexter, MD	Samuel Steerman, MD<br>Animesh Rathore, MD<br>Richard DeMasi, MD<br>Gordon Stokes, MD<br>Scott McEnroe, MD

University Surgical Associates	Chattanooga, TN	Charles Joels, MD	Mark Fugate, MD<br>Michael Greer, MD<br>Larry Richard Sprouse, MD<br>Jeff Horn, MD
Christie Clinic Vein and Vascular Center	Champaign, IL	Syed Hussain, MD<br>Nikolaos Karagiorgos, MD	Jennifer Ash, MD
Vascular Institute of Virginia	Woodbridge, VA	Sandeep Bagla, MD<br>Rachel Piechowiak, MD	
Richmond Vascular Center	North Chesterfield, VA	Jeffrey Hull, MD	
Dialysis Access Institute	Orangeburg, SC	Mark London, MD<br>John Ross, MD	Jackson Ewart, MD<br>Jalal Hakmei, MD<br>Mohamed Sheta, MD
North Carolina Nephrology	Raleigh, NC	Jeffrey Hoggard, MD	Karn Gupta, MD<br>Sejan Patel, MD
Florida Research Network LLC	Gainesville, FL	Bret Wiechmann, MD	Wesley Mann, MD
Capital Nephrology Medical Group	Sacramento, CA	Naveen Atray, MD	Rohit Kashyap, MD<br>Karthik Ramani, MD

SKI Vascular Center	Tempe, AZ	Randy Cooper, MD	Aslam Pervez, MD<br>Umar Waheed, MD
Dallas Nephrology Associates	Plano, TX	Neghae Mawla, MD	Steven Beathard, MD
Coastal Vascular and Interventional	Pensacola, FL	Fernando Kafie, MD	Huey McDaniel, MD
Clinical Site – Japan	Location	Principal Investigator	Sub-Investigator
Kansai Rosai Hospital	Amagasaki	Kotaro Suemitsu, MD	Yuki Matsuoka, MD<br>Naomi Ota, MD<br>Kanako Oka, MD<br>Saho Kawanishi, MD
Shonan Kamakura General Hospital	Kamakura	Naoko Isogai, MD<br>Hidemitsu Ogino, MD	Hidemitsu Ogino, MD<br>Katsunori Miyake, MD<br>Rai Shimoyama, MD<br>Naoko Isogai, MD<br>Jun Kawachi, MD<br>Takaaki Murata, MD<br>Nao Kume, MD<br>Yuto Igarashi, MD<br>Yuma Sunou, MD<br>Sumi Hidaka, MD

			Kunihiro Ishioka, MD
Kishiwada Tokushukai Hospital	Kishiwada	Masahiko Fujihara, MD	Yoshiaki Yokoi, MD<br>Akihiro Higashimori, MD<br>Nobuyuki Morioka, MD<br>Shinji Shiotani, MD<br>Keisuke Fukuda, MD<br>Tomofumi Tsukizawa, MD<br>Kensuke Kuwabara, MD<br>Yoshiki Matsuo, MD<br>Yuma Tanabe, MD
Shizuoka General Hospital	Shizuoka	Masaaki Murakami, MD	Noriko Mori, MD<br>Kiyoshi Mori, MD<br>Satoshi Tanaka, MD<br>Ken Matsuo, MD<br>Takao Okawa, MD<br>Shunsuke Okamura, MD<br>Yu Soma, MD<br>Yoshihiro Yamamoto, MD<br>Shota Kimura, MD<br>Yuki Ito, MD<br>Akira Sugawara, MD

			Kenta Ito, MD<br>Ryo Yamada, MD<br>Yoko Matsuo, MD<br>Kakuya Hagiwara, MD
Tokyo Women's Hospital	Shinjuku-ku	Kazuhiro Iwadoh, MD<br>Ichiro Nakajima, MD<br>Shohei Fuchinoue, MD	Sachiko Hirotani, MD<br>Kotaro Kai, MD<br>Yuichi Ogawa, MD<br>Katsuyuki Miki, MD<br>Takeshi Hachisuka, MD<br>Sayaka Morita, MD<br>Akira Kondo, MD<br>Kazuhiro Iwadoh, MD
Saitama Medical Center Saitama Medical University	Kawagoe	Tomonari Ogawa, MD	Hajime Hasegawa, MD<br>Toru Hida, MD<br>Taisuke Shimizu, MD<br>Nozomi Abe, MD<br>Tatsuro Sano, MD<br>Kunihiko Yasuda, MD<br>Tota Kiba, MD<br>Yoshimi Okada, MD<br>Koki Ogawa, MD<br>Hiroaki Hara, MD

			Kento Hirose, MD<br>Yuichiro Kawai, MD
Clinical Site – New Zealand	Location	Principal Investigator	Sub-Investigator
Auckland City Hospital	Grafton	Andrew Holden, MD	Brendan Buckley, MD<br>Brigid Connor, MD<br>Stephen Merrilees, MD<br>David Semple, MD<br>Andrew Hill, MD
Capital & Coast District Health Board	Wellington	Janaka Kesara Wickremesekera, MD (Kes Wicks)	Richard Evans, MD<br>Lupe Taumoepeau, MD<br>Anantha Narayanan, MD<br>Irina Baimatova, MD
