# Supplementary material for: Vessel and balloon sizing in the IN.PACT AV access trial: post-hoc analysis of procedural characteristics and outcomes
Source: CVIR Endovasc. 2026 Feb 14;9:17. doi: 10.1186/s42155-026-00650-6 (PMC12906498; doi:10.1186/s42155-026-00650-6)
Supplement: Supplementary file 2 — Supplementary Material 2: Table S2. 36-month target lesion primary patency Kaplan-Meier analyses and 95% confidence intervals by balloon size [file 42155_2026_650_MOESM2_ESM.pdf]

Supplemental Table 2 – Confidence Intervals of Kaplan-Meier analyses

36-month TLPP KM Estimates and 95% confidence intervals	IN.PACT AV DCB	Standard PTA	Log-Rank P value
**Stratified by Core Lab RVD Median**			
**RVD < 7.17 mm, Figure 3a, 4b**	**N= 84 Participants**	**N= 81 Participants**	
TLPP KM Estimate	42.2%	28.6%	0.024
95% CI	26.0%, 57.6%	12.7%, 46.8%	
**RVD ≥ 7.17 mm, Figure 3b, 4b**	**N= 86 Participants**	**N = 79 Participants**	
TLPP KM Estimate	43.9%	28.7%	0.011
95% CI	25.9%, 60.5%	13.6%, 45.8%	
**Stratified by Site Reported RVD Median**			
**RVD < 7.0 mm, Figure 4a**	**N= 85 Participants**	**N= 72 Participants**	
TLPP KM Estimate	43.2%	23.1%	0.002
95% CI	27.6%, 57.8%	7.2%, 44.2%	
**RVD ≥ 7.0 mm, Figure 4a**	**N= 85 Participants**	**N = 88 Participants**	
TLPP KM Estimate	42.5%	34.4%	0.06
95% CI	23.6%, 60.2%	18.8%, 50.6%	
**Stratified by Site Reported Balloon Size vs Core Lab RVD**			
**Undersized by at least 0.5 mm, Figure 5a**	**N= 66 Participants**	**N= 62 Participants**	
TLPP KM Estimate	33.2%	22.9%	0.09
95% CI	14.8%, 52.9%	8.3%, 41.8%	
**Insize by within 0.5 mm, Figure 5b**	**N= 61 Participants**	**N= 50 Participants**	
TLPP KM Estimate	56.2%	31.5%	0.001
95% CI	36.3%, 72.0%	12.9%, 52.1%	
**Oversized by at least 0.5 mm, Figure 5c**	**N= 43 Participants**	**N= 48 Participants**	
TLPP KM Estimate	33.0%	33.6%	0.53
95% CI	12.0%, 55.9%	10.4%, 59.2%	
**Stratified by Site Reported Balloon Diameter Median**			
**Ballon Dimeter < 7.0 mm, Supplemental Figure 4a, 5**	**N= 78 Participants**	**N= 70 Participants**	
TLPP KM Estimate	43.4%	24.9%	0.003
95% CI	26.2%, 59.4%	7.7%, 47.0%	
**Balloon ≥ 7.0 mm, Supplemental Figure 4a, 5**	**N= 92 Participants**	**N = 90 Participants**	
TLPP KM Estimate	42.4%	31.5%	0.0499
95% CI	25.6%, 58.3%	17.1%, 46.9%	

CI, confidence interval; DCB, drug-coated balloon; KM, Kaplan-Meier; PTA, percutaneous transluminal angioplasty; RVD, reference vessel diameter; TLPP, target lesion primary patency
