# Supplementary material for: Vessel and balloon sizing in the IN.PACT AV access trial: post-hoc analysis of procedural characteristics and outcomes
Source: CVIR Endovasc. 2026 Feb 14;9:17. doi: 10.1186/s42155-026-00650-6 (PMC12906498; doi:10.1186/s42155-026-00650-6)
Supplement: Supplementary file 3 — Supplementary Material 3: Table S3. Baseline demographics + procedural characteristics by balloon size [file 42155_2026_650_MOESM3_ESM.pdf]

Supplemental Table 3 – Baseline demographics and procedural characteristics by balloon size

| Baseline participant and lesion characteristics[a] | Balloon diameter <7 mm | | | | Balloon diameter ≥7 mm | | | |
|---|---|---|---|---|---|---|---|---|
| | IN.PACT AV DCB N=78 | Standard PTA N=70 | Total N=148 | p-value[b] | IN.PACT AV Access DCB N=92 | Standard PTA N=90 | Total N=182 | p-value [c] |
| Age (yrs) | 68.3 ± 12.3 (78) | 67.3 ± 12.6 (70) | 67.8 ± 12.4 (148) | 0.613 | 63.7 ± 13.5 (92) | 64.1 ± 13.9 (90) | 63.9 ± 13.7 (182) | 0.826 |
| Male | 62.8% (49/78) | 67.1% (47/70) | 64.9% (96/148) | 0.609 | 68.5% (63/92) | 60.0% (54/90) | 64.3% (117/182) | 0.279 |
| Hypertension | 87.2% (68/78) | 94.3% (66/70) | 90.5% (134/148) | 0.168 | 94.6% (87/92) | 94.4% (85/90) | 94.5% (172/182) | 1.000 |
| Hyperlipidemia | 59.0% (46/78) | 48.6% (34/70) | 54.1% (80/148) | 0.248 | 50.0% (46/92) | 55.6% (50/90) | 52.7% (96/182) | 0.462 |
| Diabetes Mellitus | 65.4% (51/78) | 74.3% (52/70) | 69.6% (103/148) | 0.284 | 60.9% (56/92) | 64.4% (58/90) | 62.6% (114/182) | 0.648 |
| Renal Insufficiency | 100.0% (78/78) | 100.0% (70/70) | 100.0% (148/148) | > 0.999 | 100.0% (92/92) | 100.0% (90/90) | 100.0% (182/182) | > 0.999 |
| Carotid Artery Disease | 2.6% (2/78) | 7.1% (5/70) | 4.7% (7/148) | 0.256 | 5.4% (5/92) | 10.0% (9/90) | 7.7% (14/182) | 0.278 |
| Congestive Heart Failure | 19.2% (15/78) | 27.1% (19/70) | 23.0% (34/148) | 0.328 | 26.1% (24/92) | 22.2% (20/90) | 24.2% (44/182) | 0.605 |
| Coronary Heart Disease | 39.7% (31/78) | 44.3% (31/70) | 41.9% (62/148) | 0.619 | 32.6% (30/92) | 34.4% (31/90) | 33.5% (61/182) | 0.875 |
| Peripheral Artery Disease | 23.1% (18/78) | 14.3% (10/70) | 18.9% (28/148) | 0.210 | 16.3% (15/92) | 15.7% (14/89) | 16.0% (29/181) | 1.000 |
| Current Smoker | 12.8% (10/78) | 14.3% (10/70) | 13.5% (20/148) | 0.814 | 9.8% (9/92) | 17.8% (16/90) | 13.7% (25/182) | 0.135 |
| Former Smoker | 38.5% (30/78) | 28.6% (20/70) | 33.8% (50/148) | 0.226 | 35.9% (33/92) | 27.8% (25/90) | 31.9% (58/182) | 0.268 |
| Lesion Type | | | | 0.858 | | | | 0.749 |

| | | | | | | | | |
|---|---|---|---|---|---|---|---|---|
| De Novo | 30.8% (24/78) | 28.6% (20/70) | 29.7% (44/148) | | 29.3% (27/92) | 32.2% (29/90) | 30.8% (56/182) | |
| Restenotic | 69.2% (54/78) | 71.4% (50/70) | 70.3% (104/148) | | 70.7% (65/92) | 67.8% (61/90) | 69.2% (126/182) | |
| Lesion classification | | | | 0.389 | | | | 0.236 |
| Single | 79.5% (62/78) | 85.7% (60/70) | 82.4% (122/148) | | 85.9% (79/92) | 92.2% (83/90) | 89.0% (162/182) | |
| Tandem | 20.5% (16/78) | 14.3% (10/70) | 17.6% (26/148) | | 14.1% (13/92) | 7.8% (7/90) | 11.0% (20/182) | |
| Target Lesion Location | | | | 0.587 | | | | 0.215 |
| Anastomosis | 51.3% (40/78) | 48.6% (34/70) | 50.0% (74/148) | | 4.3% (4/92) | 6.7% (6/90) | 5.5% (10/182) | |
| Arterial Inflow | 1.3% (1/78) | 7.1% (5/70) | 4.1% (6/148) | | 3.3% (3/92) | 2.2% (2/90) | 2.7% (5/182) | |
| Cephalic Arch | 3.8% (3/78) | 5.7% (4/70) | 4.7% (7/148) | | 29.3% (27/92) | 35.6% (32/90) | 32.4% (59/182) | |
| In Cannulation Zone | 3.8% (3/78) | 4.3% (3/70) | 4.1% (6/148) | | 23.9% (22/92) | 10.0% (9/90) | 17.0% (31/182) | |
| Swing Point | 6.4% (5/78) | 5.7% (4/70) | 6.1% (9/148) | | 9.8% (9/92) | 8.9% (8/90) | 9.3% (17/182) | |
| Venous Outflow | 33.3% (26/78) | 28.6% (20/70) | 31.1% (46/148) | | 29.3% (27/92) | 36.7% (33/90) | 33.0% (60/182) | |
| AVF Type | | | | 0.818 | | | | 0.817 |
| Radiocephalic | 83.3% (65/78) | 78.6% (55/70) | 81.1% (120/148) | | 22.8% (21/92) | 27.8% (25/90) | 25.3% (46/182) | |
| Brachiocephalic | 12.8% (10/78) | 14.3% (10/70) | 13.5% (20/148) | | 56.5% (52/92) | 53.3% (48/90) | 54.9% (100/182) | |
| Brachiobasilic | 1.3% (1/78) | 2.9% (2/70) | 2.0% (3/148) | | 17.4% (16/92) | 14.4% (13/90) | 15.9% (29/182) | |
| Other | 2.6% (2/78) | 4.3% (3/70) | 3.4% (5/148) | | 3.3% (3/92) | 4.4% (4/90) | 3.8% (7/182) | |

| | | | | | | | | |
|---|---|---|---|---|---|---|---|---|
| Previous peripheral revascularization | 71.8% (56/78) | 75.7% (53/70) | 73.6% (109/148) | 0.709 | 76.1% (70/92) | 74.4% (67/90) | 75.3% (137/182) | 0.864 |
| Time since AVF creation (years)[d] | 3.0 ± 3.0 (78) | 3.7 ± 4.7 (70) | 3.4 ± 3.9 (148) | 0.261 | 3.4 ± 3.0 (92) | 3.3 ± 2.9 (90) | 3.3 ± 2.9 (182) | 0.861 |
| Time on hemodialysis (years) [e] | 4.6 ± 6.1 (78) | 4.9 ± 6.4 (70) | 4.7 ± 6.2 (148) | 0.833 | 4.1 ± 4.0 (92) | 3.6 ± 4.0 (89) | 3.9 ± 4.0 (181) | 0.432 |
| Total lesion length (mm) | 56.0 ± 26.5 (78) | 50.1 ± 25.7 (70) | 53.2 ± 26.2 (148) | 0.175 | 39.2 ± 27.2 (92) | 32.1 ± 23.0 (90) | 35.7 ± 25.4 (182) | 0.059 |
| Pre-procedure stenosis (percent) | 83.9 ± 12.0 (78) | 81.2 ± 13.7 (70) | 82.6 ± 12.9 (148) | 0.205 | 77.2 ± 11.5 (92) | 77.6 ± 11.7 (90) | 77.4 ± 11.6 (182) | 0.814 |

[a] % (counts/sample size) or mean ± standard deviation (sample size)

[b] P values in this column are comparing DCB to PTA outcomes in those participants with reference vessel diameters $<7.17$ mm

[c] P values in this column are comparing DCB to PTA outcomes in those participants with reference vessel diameters $\geq 7.17$ mm

[d]24 participants had partial dates and their years since AVF creation were calculated based on the imputed dates using the middle of the month or middle of the year.

[e]29 participants had partial dates and their years of hemodialysis history were calculated based on the imputed dates using the middle of the month or middle of the year.
