# Supplementary material for: Vessel and balloon sizing in the IN.PACT AV access trial: post-hoc analysis of procedural characteristics and outcomes
Source: CVIR Endovasc. 2026 Feb 14;9:17. doi: 10.1186/s42155-026-00650-6 (PMC12906498; doi:10.1186/s42155-026-00650-6)
Supplement: Supplementary file 4 — Supplementary Material 4: Table S4. Summary of guideline recommendations about vessel sizing in the AV space [file 42155_2026_650_MOESM4_ESM.pdf]

Supplemental Table 4 – Summary of guideline recommendations about vessel sizing in the AV space

Guideline Name	Recommendation about vessel sizing
ERA-EDTA 2019 [15]	No mention of balloon sizing
ESVS 2018 [16]	No mention of balloon sizing
ACR Appropriateness Criteria 2023 [18]	No mention of balloon sizing
Brazil 2023 [19]	No mention of balloon sizing
SVS 2008 [20]	No mention of balloon sizing
Spanish 2017 [17]	No mention of balloon sizing
KDOQI 2006 [14]	Define reference vessel diameter as “the diameter of the immediately upstream or downstream normal vessel.”
KDOQI 2019 [7]	No mention of balloon sizing
JSDT 2011 [21]	Recommend using ‘the exact-size balloon [for] stenosis close to the arteriovenous anastomosis’
Kidney Health Initiative Clinical Trial Endpoints Recommendations 2018 [22]	includes a definition for normal vessel diameter: “The ‘normal’ graft or vessel immediately upstream or downstream from the lesion, whichever is smallest, should serve as the reference vessel.”

UK Consensus Guidelines 2021 [23]	Mention upsizing and prolonged inflation, as well as use of ultra-high pressure balloons, if first-line treatment fails
