# Supplementary material for: Vessel and balloon sizing in the IN.PACT AV access trial: post-hoc analysis of procedural characteristics and outcomes
Source: CVIR Endovasc. 2026 Feb 14;9:17. doi: 10.1186/s42155-026-00650-6 (PMC12906498; doi:10.1186/s42155-026-00650-6)
Supplement: Supplementary file 5 — Supplementary Material 5 Figure S1. Distribution of site-reported balloon diameter [file 42155_2026_650_MOESM5_ESM.pdf]

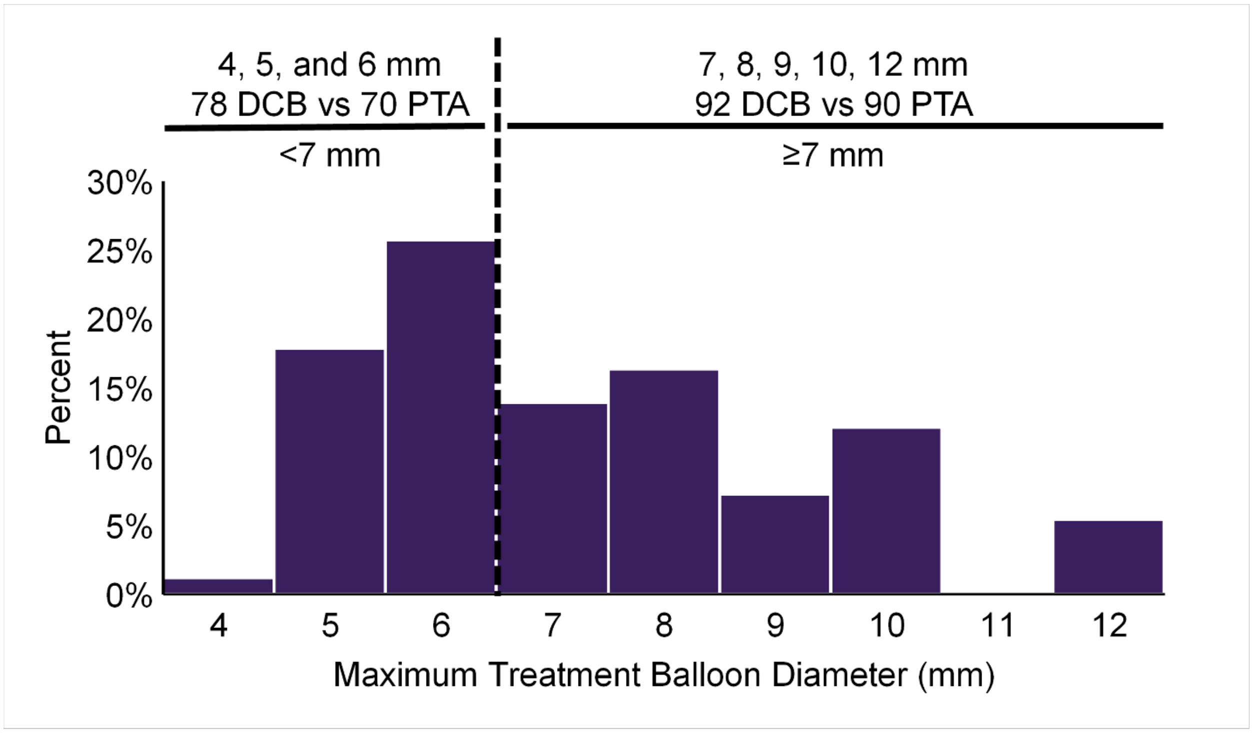

Supplemental Figure 1 – Distribution of site-reported balloon diameter

DCB, drug-coated balloon; PTA, percutaneous transluminal angioplasty
