# Supplementary material for: Vessel and balloon sizing in the IN.PACT AV access trial: post-hoc analysis of procedural characteristics and outcomes
Source: CVIR Endovasc. 2026 Feb 14;9:17. doi: 10.1186/s42155-026-00650-6 (PMC12906498; doi:10.1186/s42155-026-00650-6)
Supplement: Supplementary file 6 — Supplementary Material 6: Figure S2. Site-reported balloon size compared to core lab adjudicated reference vessel diameter [file 42155_2026_650_MOESM6_ESM.pdf]

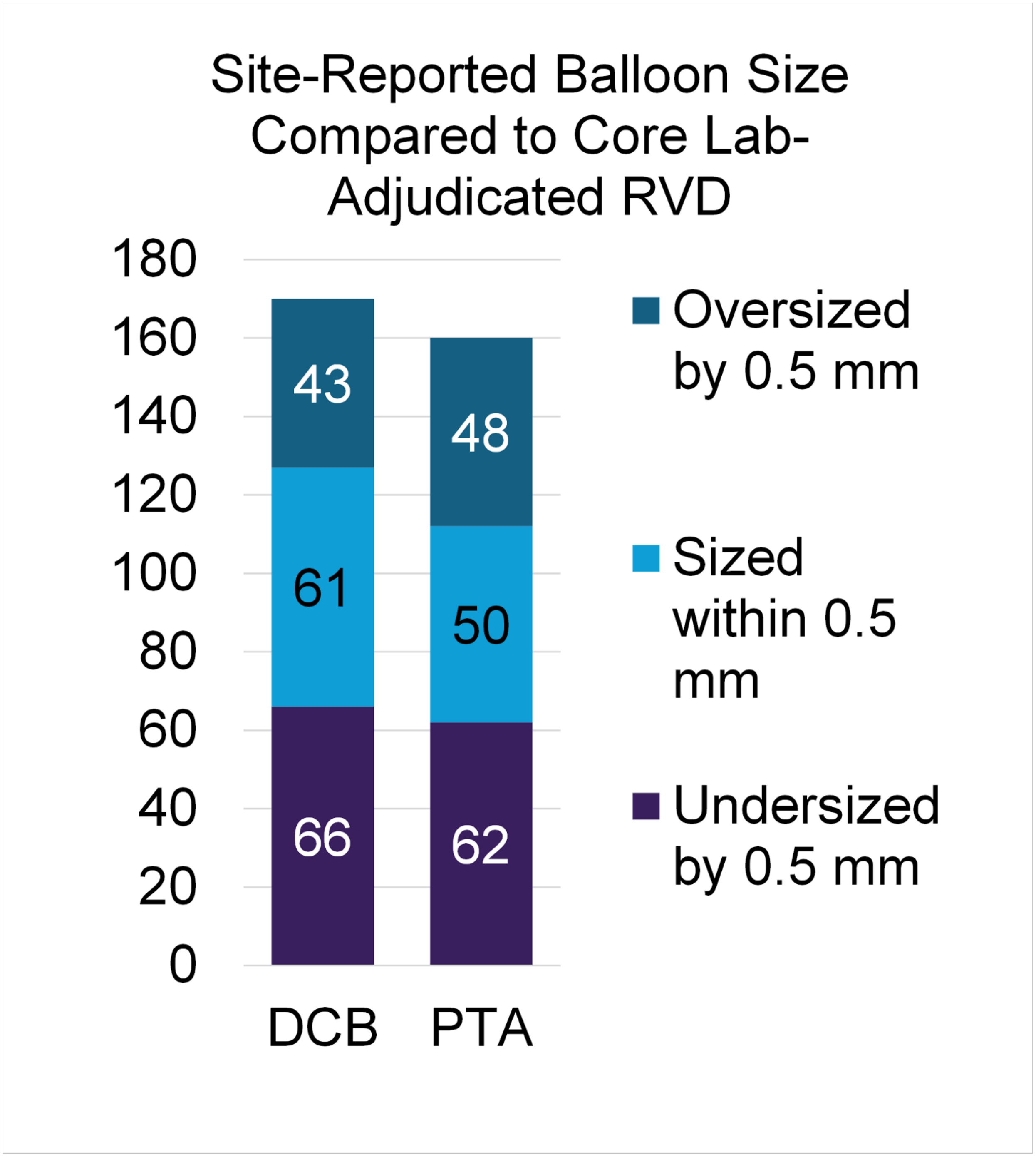

Supplemental Figure 2 – Site-reported balloon size compared to core lab adjudicated reference vessel diameter
