# Supplementary material for: Vessel and balloon sizing in the IN.PACT AV access trial: post-hoc analysis of procedural characteristics and outcomes
Source: CVIR Endovasc. 2026 Feb 14;9:17. doi: 10.1186/s42155-026-00650-6 (PMC12906498; doi:10.1186/s42155-026-00650-6)
Supplement: Supplementary file 7 — Supplementary Material 7 Figure S3. Forest plot of target lesion primary patency through 36 months by core lab adjudicated median final reference vessel diameter [file 42155_2026_650_MOESM7_ESM.pdf]

Subgroups (N DCB/N PTA)	IN.PACT AV DCB*	Standard PTA*	Hazard Ratio [95% CI]
6 Months			
RVD < 7.17 mm (84/81)	81.4% (15)	67.9% (24)	0.534 [0.280, 1.019]
RVD ≥ 7.17 mm (86/79)	92.0% (6)	70.3% (22)	0.231 [0.094, 0.571]
12 Months			
RVD < 7.17 mm (84/81)	63.6% (29)	46.0% (40)	0.555 [0.344, 0.896]
RVD ≥ 7.17 mm (86/79)	66.8% (22)	47.1% (37)	0.467 [0.276, 0.792]
24 Months			
RVD < 7.17 mm (84/81)	53.8% (36)	40.1% (44)	0.607 [0.390, 0.945]
RVD ≥ 7.17 mm (86/79)	49.2% (32)	31.9% (45)	0.549 [0.349, 0.864]
36 Months			
RVD < 7.17 mm (84/81)	42.2% (42)	28.6% (49)	0.623 [0.412, 0.942]
RVD ≥ 7.17 mm (86/79)	43.9% (34)	28.7% (46)	0.566 [0.363, 0.882]

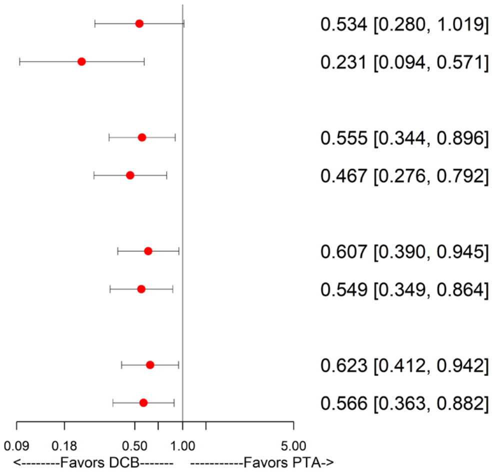

Supplemental Figure 3 – Forest plot of target lesion primary patency through 36 months by core lab adjudicated median final reference vessel diameter

DCB, drug-coated balloon; PTA, percutaneous transluminal angioplasty; RVD, reference vessel diameter. All events were adjudicated by the independent and blinded Clinical Events Committee. Target lesion primary patency is defined as freedom from clinically-driven target lesion revascularization or access circuit thrombosis. An event was adjudicated as a clinically-driven target lesion revascularization if the target lesion had a ≥50% diameter stenosis (per angiographic core lab assessment) in the presence of clinical or physiologic abnormalities that indicate dialysis access dysfunction or a ≥70% stenosis without the presence of clinical or physiologic abnormalities indicating dialysis access dysfunction.
