# Supplementary material for: Vessel and balloon sizing in the IN.PACT AV access trial: post-hoc analysis of procedural characteristics and outcomes
Source: CVIR Endovasc. 2026 Feb 14;9:17. doi: 10.1186/s42155-026-00650-6 (PMC12906498; doi:10.1186/s42155-026-00650-6)
Supplement: Supplementary file 8 — Supplementary Material 8: Figure S4. Kaplan-Meier analysis of target lesion primary patency through 36 months by balloon diameter (a, <7 mm; b ≥7 mm) [file 42155_2026_650_MOESM8_ESM.pdf]

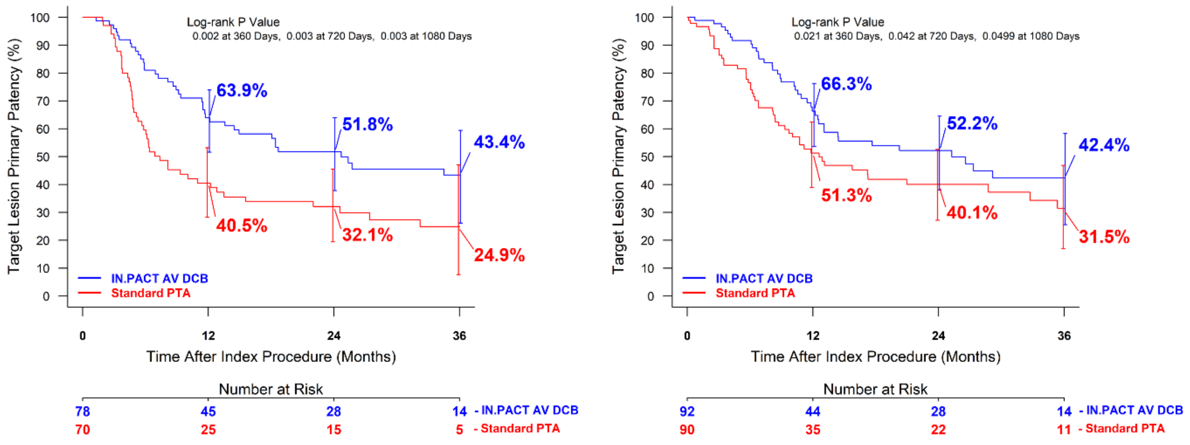

Supplemental Figure 4a/b: Kaplan-Meier analysis of target lesion primary patency through 36 months by balloon diameter (a, <7 mm; b ≥7 mm)

DCB, drug-coated balloon; PTA, percutaneous transluminal angioplasty. All events were adjudicated by the independent and blinded Clinical Events Committee. Target lesion primary patency is defined as freedom from clinically-driven target lesion revascularization or access circuit thrombosis. An event was adjudicated as a clinically-driven target lesion revascularization if the target lesion had a ≥50% diameter stenosis (per angiographic core lab assessment) in the presence of clinical or physiologic abnormalities that indicate dialysis access dysfunction or a ≥70% stenosis without the presence of clinical or physiologic abnormalities indicating dialysis access dysfunction.
