# Supplementary material for: Vessel and balloon sizing in the IN.PACT AV access trial: post-hoc analysis of procedural characteristics and outcomes
Source: CVIR Endovasc. 2026 Feb 14;9:17. doi: 10.1186/s42155-026-00650-6 (PMC12906498; doi:10.1186/s42155-026-00650-6)
Supplement: Supplementary file 10 — Supplementary Material 10: Figure S6. Forest plot of target lesion primary patency through 36 months by balloon diameter [file 42155_2026_650_MOESM10_ESM.pdf]

Subgroups (N DCB/N PTA)	IN.PACT AV DCB*	Standard PTA*	Hazard Ratio [95% CI]
6 Months			
Balloon Diameter <7mm (78/70)	81.0% (14)	59.5% (26)	0.400 [0.209, 0.768]
Balloon Diameter ≥7mm (92/90)	91.7% (7)	76.5% (20)	0.327 [0.138, 0.775]
12 Months			
Balloon Diameter <7mm (78/70)	63.9% (26)	40.5% (38)	0.454 [0.275, 0.749]
Balloon Diameter ≥7mm (92/90)	66.3% (25)	51.3% (39)	0.558 [0.338, 0.923]
24 Months			
Balloon Diameter <7mm (78/70)	51.8% (34)	32.1% (43)	0.508 [0.323, 0.798]
Balloon Diameter ≥7mm (92/90)	52.2% (34)	40.1% (46)	0.634 [0.407, 0.988]
36 Months			
Balloon Diameter <7mm (78/70)	43.4% (38)	24.9% (46)	0.521 [0.339, 0.803]
Balloon Diameter ≥7mm (92/90)	42.4% (38)	31.5% (49)	0.657 [0.430, 1.004]

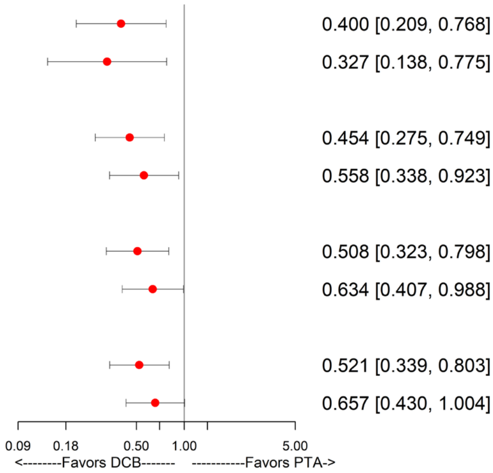

Supplemental Figure 6 – Forest plot of target lesion primary patency through 36 months by balloon diameter

DCB, drug-coated balloon; PTA, percutaneous transluminal angioplasty. All events were adjudicated by the independent and blinded Clinical Events Committee. Target lesion primary patency is defined as freedom from clinically-driven target lesion revascularization or access circuit thrombosis. An event was adjudicated as a clinically-driven target lesion revascularization if the target lesion had a ≥50% diameter stenosis (per angiographic core lab assessment) in the presence of clinical or physiologic abnormalities that indicate dialysis access dysfunction or a ≥70% stenosis without the presence of clinical or physiologic abnormalities indicating dialysis access dysfunction.
